# Characterization of an Arabidopsis Defensin-like Gene Conferring Resistance against Nematodes

**DOI:** 10.3390/plants11030280

**Published:** 2022-01-21

**Authors:** Abdalmenem I. M. Hawamda, Susanne Reichert, Muhammad Amjad Ali, Muhammad Amjad Nawaz, Tina Austerlitz, Patricia Schekahn, Amjad Abbas, Raimund Tenhaken, Holger Bohlmann

**Affiliations:** 1 Institute of Plant Protection, Department of Crop Sciences, University of Natural Resources and Life Sciences, 1180 Vienna, Austria; a.hawamdeh@ptuk.edu.ps (A.I.M.H.); reichert.sue@gmail.com (S.R.); amjad.ali@uaf.edu.pk (M.A.A.); tina.austerlitz@boku.ac.at (T.A.); patriciaschekahn@web.de (P.S.); amjad.abbas@uaf.edu.pk (A.A.); 2 Department of Agricultural Biotechnology, Faculty of Agricultural Science and Technology, Palestine Technical University-Kadoorie (PTUK), Tulkarm P.O. Box 7, Palestine; 3 Department of Plant Pathology, University of Agriculture, Faisalabad 38040, Pakistan; 4 Centre of Agricultural Biochemistry and Biotechnology, University of Agriculture, Faisalabad 38040, Pakistan; 5 Siberian Federal Scientific Centre of Agrobiotechnology, Russian Academy of Sciences, 630501 Krasnoobsk, Russia; amjad_ucauos@yahoo.com; 6 Laboratory of Supercritical Fluid Research and Application in Agrobiotechnology, The National Research Tomsk State University, 36, Lenin Avenue, 634050 Tomsk, Russia; 7 Plant Physiology, University of Salzburg, 5020 Salzburg, Austria; raimund.tenhaken@sbg.ac.at

**Keywords:** Arabidopsis, *Heterodera* *schachtii*, plant defense, antimicrobial peptide

## Abstract

Arabidopsis contains 317 genes for defensin-like (DEFL) peptides. DEFLs have been grouped into different families based mainly on cysteine motifs. The DEFL0770 group contains seven genes, of which four are strongly expressed in roots. We found that the expression of these genes is downregulated in syncytia induced by the beet cyst nematode *Heterodera schachtii* as revealed by RNAseq analysis. We have studied one gene of this group, *At3g59930*, in detail. A promoter::GUS line revealed that the gene is only expressed in roots but not in other plant organs. Infection of the GUS line with larvae of *H. schachtii* showed a strong downregulation of GUS expression in infection sites as early as 1 dpi, confirming the RNAseq data. The At3g59930 peptide had only weak antimicrobial activity against *Botrytis cinerea*. Overexpression lines had no enhanced resistance against this fungus but were more resistant to *H. schachtii* infection. Our data indicate that *At3g59930* is involved in resistance to nematodes which is probably not due to direct nematicidal activity.

## 1. Introduction

Nematodes are a widespread group of animals which includes many pathogens of plants and animals. The family *Heteroderidae* comprises obligate biotrophic sedentary plant parasites. They attack mainly the roots of a variety of plants which can severely damage crop plants. One major group within the family *Heteroderidae* are the cyst nematodes [1], sedesntary endoparasites which enter the plant roots as second stage juveniles (J2 larvae). After their entrance into the roots, they establish a feeding structure which is called a syncytium [2]. The syncytium is initiated from a single root cell and grows by incorporating up to several hundred cells via local cell wall modification and degradation. The syncytium is the only nutrient source for the nematodes and thus a severe nutrient sink for the plant. Nutrients are taken up through the stylet. For that the nematodes produce feeding tubes new during each feeding cycle [3]. Adult male cyst nematodes leave their feeding site after some time to mate with females. The female cyst nematodes stay with the syncytium throughout their life and grow to such a size that they can rupture the root, while the head remains associated with the syncytium. Females produce several hundred eggs that are mostly kept within their body. Once the female dies, its body hardens and becomes the so-called cyst which can protect the eggs for many years until the next generation hatches from the cyst under favourable environmental conditions.

The development of the syncytium starts with the initial syncytial cell and is initiated through secretions of the nematode that lead to upregulation or downregulation of the expression of many plant genes [4]. Upregulated plant genes, for instance, code for expansins and cellulases which are involved in the degradation of cell walls such that additional cells can be incorporated into the growing syncytium [5,6]. But syncytial cell walls are also modified which requires the synthesis of new cell wall polysaccharides [6]. At the interface between syncytia that are associated with female nematodes and xylem vessels protuberances are produced which are supposed to be needed for the transport of water and solutes [2]. It was found that their production depends on functional *UGD2* and *UGD3* genes which encode UDP-glucose dehydrogenase [7]. In addition, the outer cell walls of the syncytium are strengthened and this also requires the production of new cell wall polysaccharides [6].

The structure and the activity of cells within the syncytium changes. Syncytial cells contain many small vacuoles instead of a large central vacuole and dense granular cytoplasm, large numbers of mitochondria, ribosomes, and proliferated endoplasmic reticulum representing high metabolic activity [8]. Nuclei and nucleoli are enlarged. They contain endoreduplicated DNA to support the high metabolic activity of the syncytium [9]. Several nematode proteins from the dorsal pharyngeal gland cell have been identified as effectors that are probably responsible for inducing the development of the syncytium [10,11,12].

In addition to nematodes, plants are constantly threatened by a large variety of other pathogens. Plants have developed a wide range of defenses which can be divided into constitutive and induced defenses. These mechanisms include the production of small molecular weight compounds, phytoanticipins and phytoalexins. Based on the recognition of pathogens with the help of resistance genes, plants can also mount a so-called hypersensitive response. This mechanism is especially effective against biotrophic pathogens [13,14]. Plants also produce a variety of pathogenesis-related (PR)-proteins [14] which include chitinases, glucanases and several families of antimicrobial peptides (AMPs). Based on sequence similarity, cysteine (Cys) motifs and disulfide bond patterns, plant AMPs have been arranged into different families, including thionins [15], plant defensins [16], lipid transfer proteins (LTP) [17], knottin-like peptides [18], hevein-type peptides [19], cyclotides [20] and snakins [21].

Plant defensins are widely distributed in the plant kingdom and are perhaps found in every plant species [22]. They are usually secreted, approximately 50 amino acids long, positively charged and cysteine-rich. Their three-dimensional structure is comprised of a single α-helix and a triple-stranded antiparallel β-sheet stabilized by four or five disulfide bonds. Many plant defensins have been shown to have antimicrobial activity against bacteria and fungi [23]. Some plant defensins are also active against insects [24], while others are involved in heavy metal tolerance [25]. Others have been reported to work as protein translation inhibitors [26], protease inhibitors [27] and ion channel blockers [28]. Expression of defensin genes in transgenic plants has been reported to increase the resistance of the plant against various pathogens [29].

Arabidopsis contains 13 plant defensin genes [30] which are either constitutively expressed in various organs or are induced after a pathogen encounter. *Pdf1.2* is for instance regulated by jasmonic acid and ethylene [31,32]. *Pdf1.1* has been shown to be involved in response to plant pathogens [33], perhaps by sequestering iron which is needed by microbial pathogens [34]. Two other plant defensins, Pdf2.5 and Pdf2.6, have been shown to chelate cadmium [35,36]. In addition to plant defensins, Arabidopsis and other plant species contain a large number of defensin-like peptides (DEFLs). For instance, 317 and 93 DEFLs have been identified in Arabidopsis and *Oryza sativa*, respectively [37]. Various activities have been reported for DEFLs, ranging from pollen tube attractants [38] to controlling pollen tube burst in maize [39]. DEFLs have been grouped into different cysteine rich protein (CRP) families based mainly on cysteine motifs [37].

Here we have studied a member of the CRP0770 group. This group of DEFLs contains 7 genes and we have shown that *At3g59930* is downregulated in syncytia induced by the beet cyst nematode *H. schachtii*. Overexpression resulted in enhanced resistance against *H. schachtii*. The encoded peptide had only low in vitro antimicrobial activity.

## 2. Results

**Figure 1:**
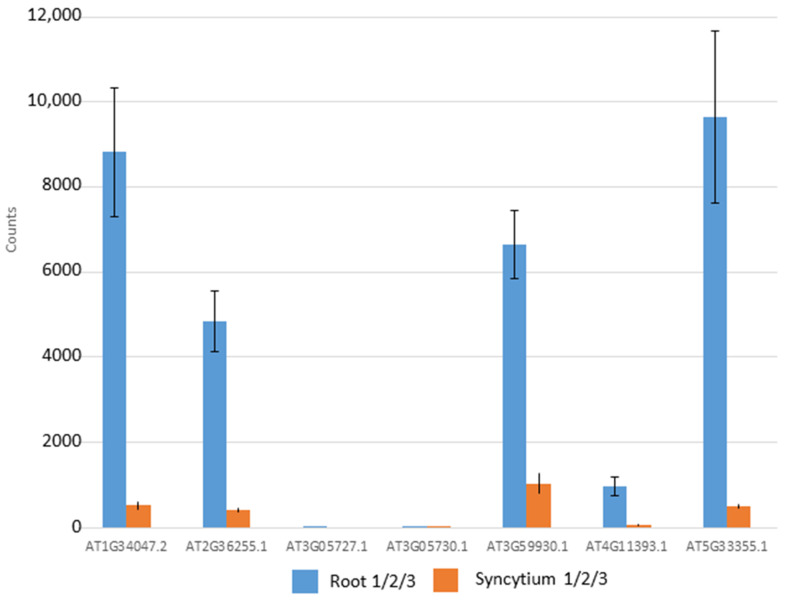
Expression of CRP0770 genes in roots and syncytia. The data shown are TPM (transcript per million) mean values from 3 RNAseq experiments each for roots and syncytia induced by the beet cyst nematode *H. schachtii*. The error bar represents standard deviation of the mean.

During a project which included RNAseq analysis of syncytia induced by the beet cyst nematode *H. schachtii* in Arabidopsis roots, we found that the expression of several genes of the CRP0770 group [37] was downregulated (Figure 1, Table S1). Only one of these genes, *At3g05730*, was included in our previous analysis of the syncytium transcriptome using Affymetrix GeneChips [40]. However, this gene [40] and the closely related *At3g05727* are expressed in roots and syncytia at a very low level (Figure 1, Table S1).

### 2.1. In Silico Characterization of CRP0770 Genes

**Figure 2:**
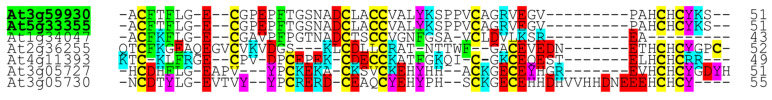
Sequence alignment of mature peptides encoded by CRP0770 genes. *At3g59930* and *A5g33355* (marked green) encode identical peptides. Sequences were aligned with Clustal Omega and gaps were introduced to align the cysteine residues (yellow). Blue marks, basic amino acids; red, acidic amino acids; green, phenylalanine; pink, tyrosin.

**Figure 3:**
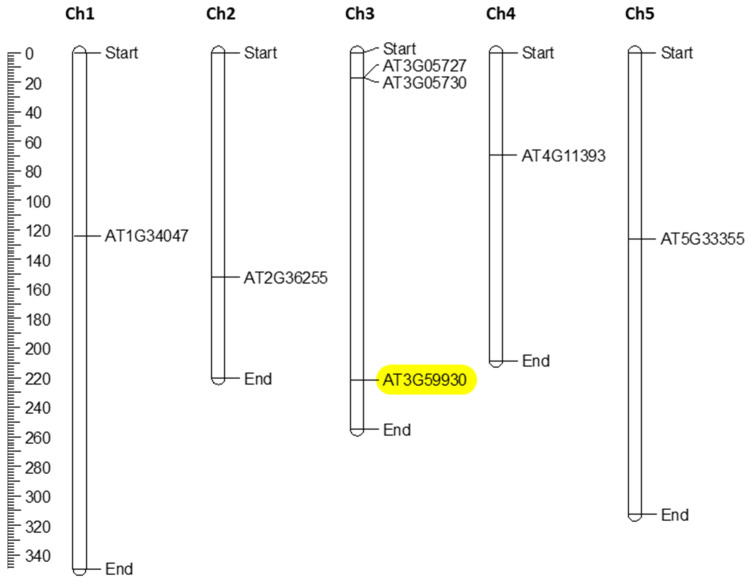
Chromosomal positioning of Arabidopsis CRP0770 genes. The scale on the left side of the figure gives the chromosomal length (in mega base pairs). The chromosomal number is shown at the top of the figure. The *At3g59930* gene is highlighted in yellow.

**Figure 4:**
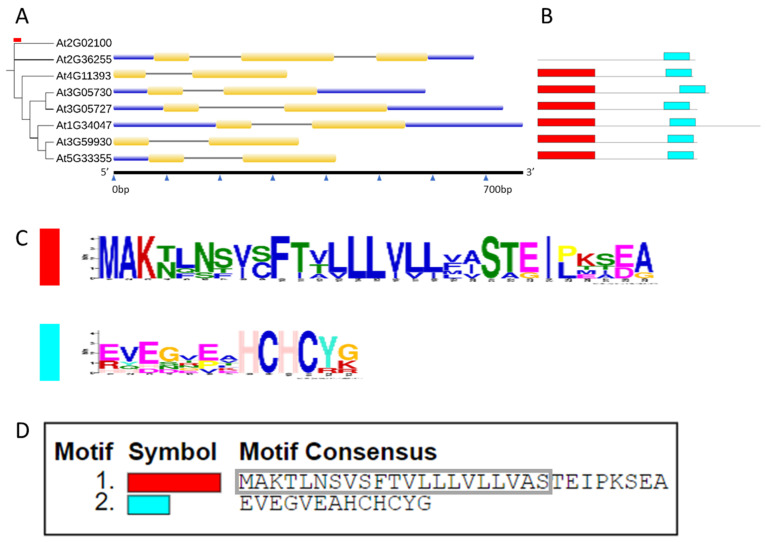
Comparison of Arabidopsis CRP0770 genes. (**A**), Phylogenetic tree and gene structure. The maximum likelihood (ML) tree was generated by the WAG + G4 model using ultrafast bootstrap with 1000 replicates in iQ-Tree web server. *At2g02100* (a plant defensin gene) was included as an out group. The red bar on the tree shows tree scale = 0.1. Exons are shown in yellow and black lines indicate introns. UTRs (5′and 3′ untranslated regions) are shown in blue. (**B**–**D**), Conserved motif analysis. (**B**), the motifs are represented with different colors, motif 1 red and motif 2 aqua blue. (**C**), sequence logos of the motifs. Conserved motif analysis found 2 motifs. Motif 1 consisted of the signal peptide and the beginning of the mature peptide. Motif 2 contained the last 2 cysteines. (**D**), consensus sequence for both motifs. Motif 1 contains the signal peptide (indicated by a grey box). Motif 2 is conserved in all genes, while motif 1 is missing in *At2g36255*.

The CRP0770 genes encode peptides with a signal peptide according to SignalP. An alignment of the mature peptides (Figure 2) shows the conservation of the cysteine residues. We used MapChart to map all seven genes on the Arabidopsis chromosomes. They were distributed across all five chromosomes with Ch 3 having three genes and Ch 1, 2, 4, and 5 having one gene each (Figure 3). The ML (maximum likelihood) tree demonstrated that *At5g33355* and *At3g59930* as well as *At3g05730* and *At3g0527* formed gene pairs. Gene structure analysis showed that the CRP0770 genes were characterized by the presence of one intron (except *At1g34047,* which had two introns) as shown in Figure 4A. Conserved motif analysis found 2 motifs. Motif 1 consisted of the signal peptide and the beginning of the mature peptide. Motif 2 contained the last 2 cysteines (Figure 4B–D).

**Figure 5:**
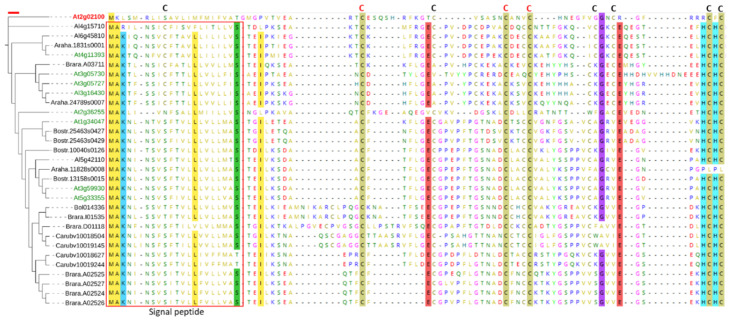
Maximum likelihood (ML) tree of homologs of *At3g59930*. The tree was constructed by aligning the peptides of homologs in MEGA v. 7.0 using the built-in MUSCLE algorithm for multiple sequence alignment. The alignment was then exported to iQTree. ModelFinder was used to find a model of substitution, which was WG + G4. The ML tree was then constructed based on the selected model with ultrafast bootstrap and 1000 replicates. *At2g02100* (a plant defensin gene) was used as an outgroup to root the tree. The red scale on the top of the tree represents the tree scale = 0.1. Red box shows the conserved signal peptide. Letters C on the top of alignment show conserved cysteine residues, with red colour indicating 100% conservation across all the entries. The highlighting with different colors represent residues that are conserved in >90% entries.

To understand the possible presence of similar peptides in other land plants, we searched against all sequenced plant genomes available at Phytozome v12.1. The BLASTp search against 85 sequenced *Viridiplantae* genomes (using the peptide sequence of *At3g59930* as a query) resulted in the identification of 23 genes belonging to six *Brassicaceae* species (Figure 5). Other DEFL gene families have been reported in other plant lineages outside *Brassicaceae* [41]. Alignment of the peptide sequences of all 30 *Brassicacea* DEFLs showed the presence of a consensus signal peptide and conserved C residues at different positions. The absence of these DEFLs from other plant lineages possibly suggests that *Brassicaceae* family members have evolved these genes very recently when the two *Brassicaceae* lineages, i.e., I and II, were derived from a common ancestor, approximately 20 million years ago [42].

### 2.2. Expression Analysis of At3g59930

**Figure 6:**
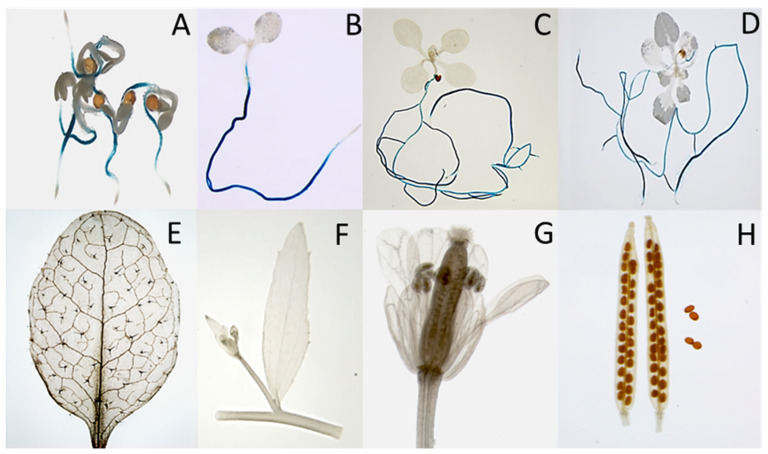
GUS expression in the *At3g59930::GUS* transgenic line. (**A**–**D**), seedlings grown on MS medium for 1, 5, 10 and 14 days. GUS expression is only visible in roots but not root tip, cotyledons or leaves. (**E**–**H**), plants grown on soil in long day. No GUS expression was found in rosette leaves (**E**), stem and cauline leaves (**F**), flowers (**G**) or siliques and seeds (**H**).

Four genes of the CRP0770 group are expressed in roots at a high level and strongly downregulated in syncytia, while *At4g11393* is expressed at approximately a tenfold lower level in roots but also downregulated in syncytia (Figure 1, Table S1). We selected *At3g59930* for a detailed analysis. Although this gene and the closely related *A5g33355* are represented with probes on the Affymetrix GeneChip, we had excluded them from our earlier analysis of the syncytium transcriptome because of too high sequence similarity [40]. To study the expression of *At3g59930* in detail we produced promoter::GUS lines. Initial analysis of 10 independent lines found that GUS expression was only visible in roots but not in other tissues. One line was selected for a detailed analysis which showed expression in roots with the exception of root tips but not in cotyledons, leaves, cauline leaves, stems, flowers, siliques and seeds (Figure 6).

**Figure 7:**
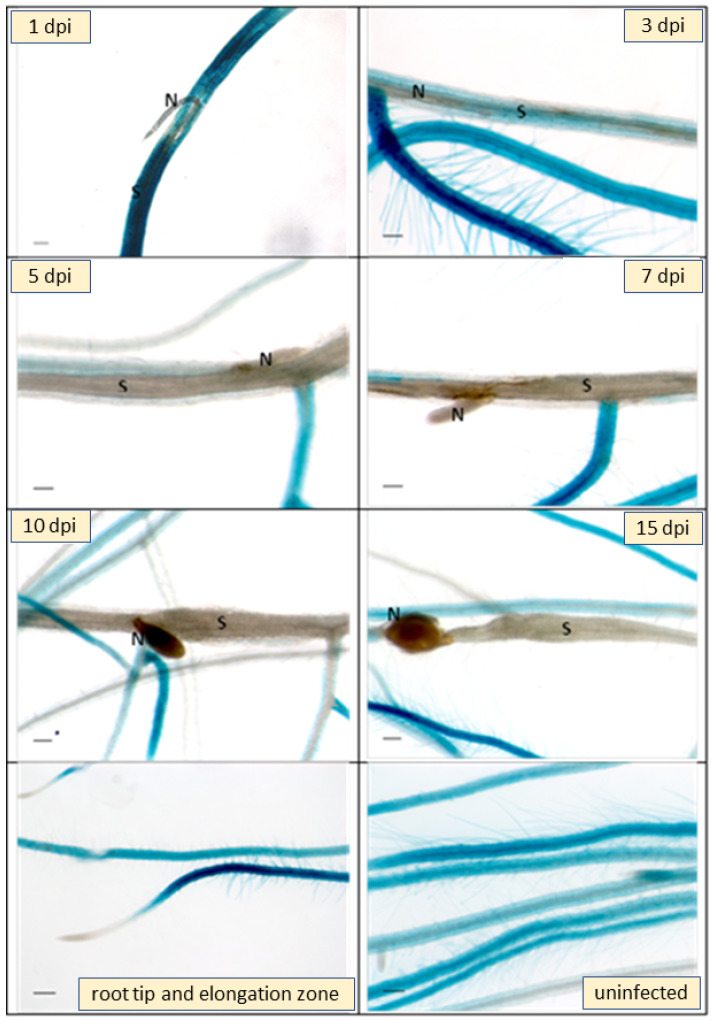
GUS expression in a *At3g59930::GUS* transgenic line infected with *H. schachtii*. Plants were grown on Knop medium. The figure shows infection sites on roots at 1, 3, 5, 7, 10 and 15 dpi (days post infection). At the bottom uninfected roots and root tips with elongation zone are shown. Downregulation of GUS expression at infection sites starts as early as 1 dpi. S, syncytium; N, nematode. Bar = 100 μm.

Infection of the GUS line with larvae of *H. schachtii* showed a downregulation of GUS expression in infection sites as early as 1 dpi (Figure 7). At 5 dpi syncytia were totally lacking GUS expression and at later stages the downregulation extended into side roots originating at the site of syncytia. Thus, GUS analysis confirmed the RNAseq data.

We used the promoter::GUS line of *At3g59930* to study the response to leaf infecting pathogens. Leaves of 4 week old plants were infected with the fungi *B. cinerea* and *A. brassicicola* by placing drops with spores on the leaves. We stained the leaves for GUS at different times after infection but did not find any staining. We also infected the leaves by infiltrating a suspension of the bacterial pathogen *P. syringae*. Again, we did not find an induction of the promoter::GUS construct (Figure S1).

There is some information available about expression of the CRP0770 genes from a microarray analysis of all Arabidopsis DEFL genes [43]. According to that study, only *At3g59930* and *At5g33355* are strongly expressed in roots (Table S2) which is in contrast to our RNAseq analysis (Table S1). We found that *At1g34047* and *At2g36255* are also expressed in roots at a high level. The study from Tesfaye et al. also reported very low expression in inflorescences and siliques but high expression in 7 day and 14 day old seedlings. The authors also studied expression after infection with *Pseudomonas syringae* and *Alternaria brassicicola* (Tables S3 and S4). They found a strong induction of *At3g59930* and *At5g33355* after infection with *A. brasscicola*. There was no induction of any of the seven CRP0770 genes after infection with *P. syringae* pv *tomato* DC3000.

### 2.3. In Vitro Antimicrobial Assays

**Figure 8:**
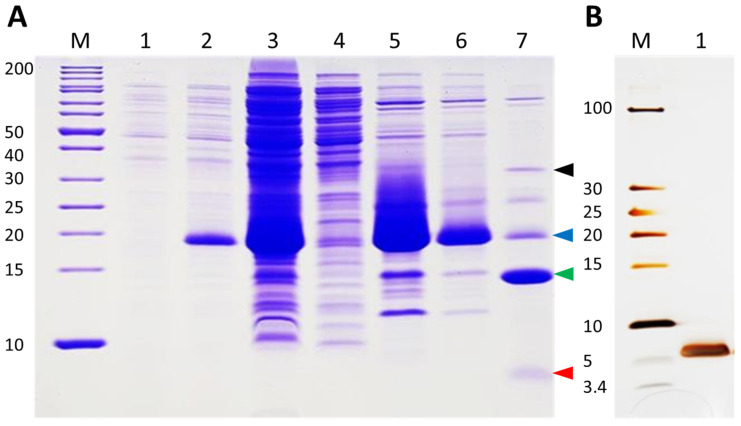
DEFL expression in *E. coli.* (**A**). SDS-PAGE (Coomassie blue stained gel) analysis of the purification steps. Lane M: marker; Lane 1: cell lysate before IPTG induction; Lance 2: cell lysate after IPTG induction; Lane 3: supernatant after sonication; Lane 4: flow-through after His-tag purification; Lane 5: first elution after His-tag purification; Lane 6: second elution after His-tag purification; Lane 7: TEV digestion of the fusion protein. Black, blue, green and red arrowheads indicate TEV protease, fusion protein, thioredoxin and At3g59930 peptide, respectively. (**B**) Silver stained gel. Lane M: marker; Lane 1: purified At3g59930 peptide after reverse phase chromatography. Size of the marker bands is given on the left side of the gels in kDa.

**Figure 9:**
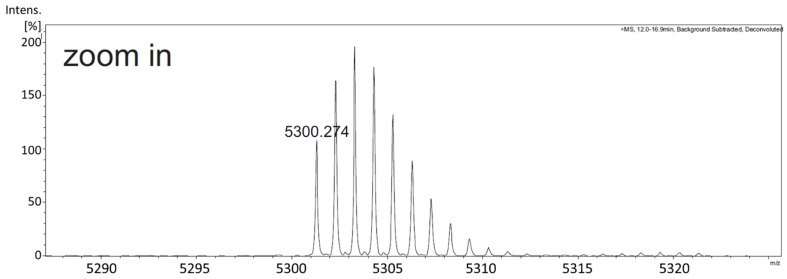
ESI-MS of purified At3g59930 peptide. Measured mass 5300.274 indicates the expected size of the At3g59930 peptide and confirmed that all four disulfide bridges were intact. ESI-MS was done by C. Grünwald-Gruber at the BOKU Core Facility Biomolecular & Cellular Analysis.

DEFL peptides have been shown to have antimicrobial activity in vitro [44]. For these in vitro tests, we produced the At3g59930 peptide in *Escherichia coli*. The coding sequence without signal peptide was fused to the TEV site of the vector pETtrx1a (Figure S2). Figure 8A shows the expression and purification of the peptide. The fusion protein is clearly visible after induction with isopropylthiogalactoside (IPTG). It was enriched after His-tag purification and most of the fusion protein could be digested with TEV protease. After TEV digestion, the At3g59930 peptide was purified with reverse phase chromatography (Figure 8B) and confirmed by ESI-MS (Figure 9). The peptide would have a theoretical mass with free cysteines of 5308.3408 Da and a theoretical mass with intact disulfide bonds of 5300.2783 Da. We measured 5300.274 Da and, thus, confirmed that all 4 disulfide bridges were intact.

**Figure 10:**
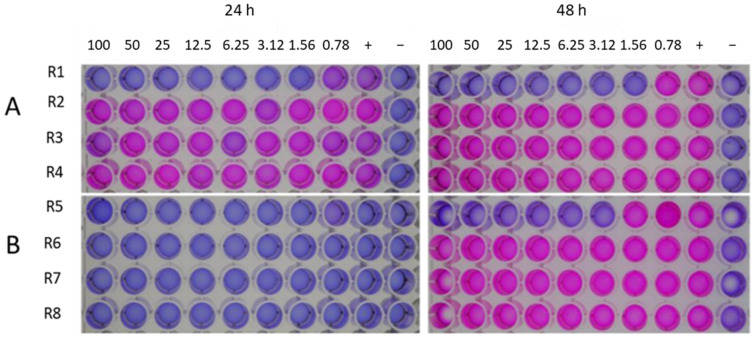
Antibacterial activity of At3g59930 peptide using resazurin assay. (**A**), against *P. syringae* and (**B**), against *A. tumefaciens* after 24 and 48 h incubation. Kanamycin was used as a control (R1 and R5) in (**A**) and (**B**), respectively. R2–R4 in (**A**) are replicates against *P. syringae*, while R6–R8 in (**B**) are the replicates against *A. tumefaciens*. Concentration in μg/mL, for both kanamycin and the At3g59930 peptide are shown on top of the figure. +, positive control (no kanamycin or At3g59930 peptide), −, negative control (only minimal medium). Results indicated that the At3g59930 peptide has no antibacterial activity.

We tested the At3g59930 peptide against 2 different bacteria, *P. syringae* pv. *Tomato* DC3000 and *Agrobacterium tumefaciens* GV3101 (Figure 10) using the resazurin assay. For *P. syringae*, growth was visible after 24 h at all concentrations, while *A. tumefaciens* took 48 h to grow. The latter was also not inhibited at any concentration of the peptide. Kanamycin controls showed clear inhibition of both bacteria at low concentrations. Thus, the At3g59930 peptide had no effect on the bacteria tested.

**Figure 11:**
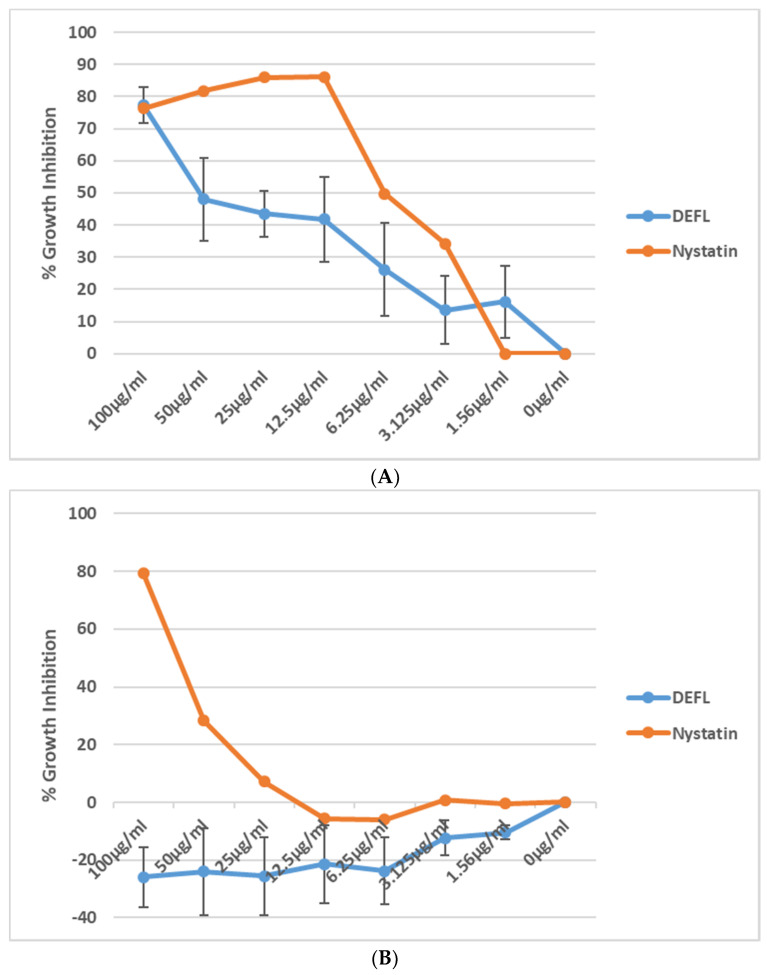
Antifungal activity of the At3g59930 peptide. (**A**), *B. cinerea* and (**B**), *F. oxysporum*. Shown is percent growth inhibition with standard deviation after 48 h against peptide concentrations ranging from 1.56 µg/mL to 100 µg/mL. The fungicide nystatin was used as a control. At3g59930 had no activity against *F. oxysporum* but was active against *B. cinerea* with an IC_50_ of approximately 50 µg/mL.

A possible antifungal effect was tested with *B. cinerea* and *F. oxysporum* f. sp. *matthiolae* using OD measurements (Figure 11). We found inhibition of *B. cinerea* with an IC_50_ of approximately 50 µg/mL. However, no effect on *F. oxysporum* was found.

### 2.4. Overexpression of At3g59930

**Figure 12:**
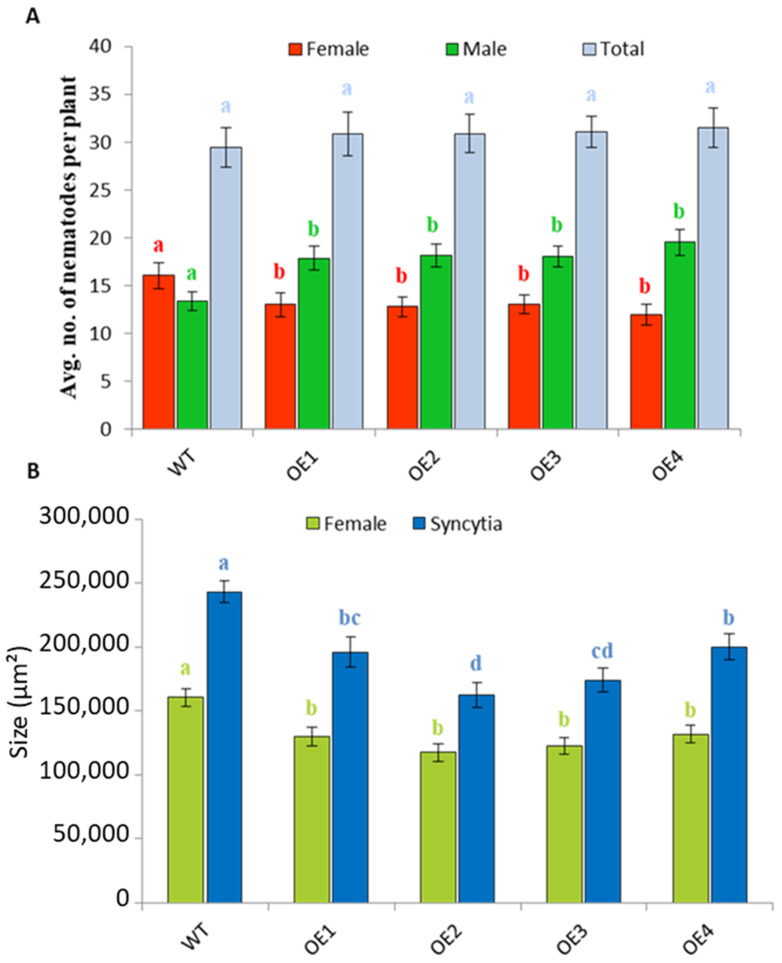
Overexpression of *At3g59930* reduced susceptibility to *H. schachtii***.** Twelve-day old seedlings were grown on Knop medium and infected with *H. schachtii* larvae. Each plant was infected with 50–60 J2 nematodes. (**A**), Male and female nematodes were counted at 15 dpi. (**B**), size of female nematodes and syncytia at 15 dpi. Mean data from three experimental replications was subjected to analysis of variance and differences among mean determined by LSD at a 5% significance level and *n* = 18 with different letters above the columns indicating significant differences. The values are means ± SE. WT, wild type; OE, overexpression line.

Downregulation of the CRP0770 genes in syncytia indicated that expression might negatively affect the development of nematodes. We therefore produced overexpression lines for *At3g59930* and selected four independent lines using RT-PCR (Figure S3). We did not detect any phenotypical differences between the overexpression lines and the wild type. The overexpression lines were infected with *H. schachtii* larvae (Figure 12). On all 4 overexpression lines, we found a significantly lower number of females but a higher number of males as compared to wild type, while the total number of nematodes was not different (Figure 12A). Overexpression also had a direct effect on syncytia and female nematodes. Both syncytia and female nematodes were significantly smaller on overexpression lines than on the wild type (Figure 12B).

**Figure 13:**
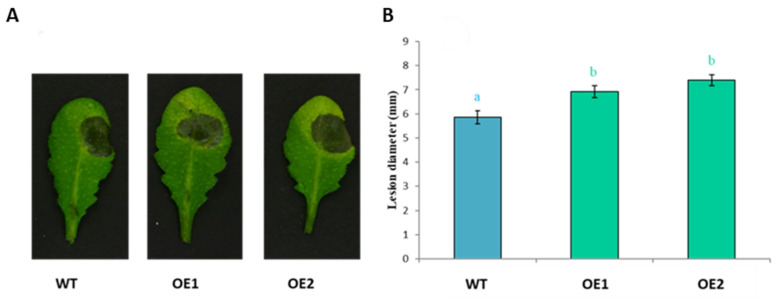
Overexpression of *At3g59930* increased susceptibility to *B. cinerea.* Overexpression lines of *At3g59930* were compared with wild type plants. Plants were grown in short day with 16 h dark and 8 h light at 24 °C for 4 weeks. Leaves were infected with 5 μL drop of *B. cinerea* spores. For each line, 4–5 plants were infected per replicate. (**A**). Representative pictures were taken at 3 dpi. (**B**). Lesion diameter in mm at 3 dpi. Mean data from three experimental replications were subjected to analysis of variance and differences among mean determined by LSD at a 5% significance level and *n* = 20. The values are means ± SE. different letters “a” and “b” indicate significant difference. WT, wild type; OE; overexpression line.

In the antimicrobial assays, we found low activity against *B. cinerea*. Therefore, two of the overexpression lines were tested against this fungus. We found that there was a small but significant increase in the susceptibility of these lines against *B. cinerea* (Figure 13).

## 3. Discussion

### 3.1. In Silico Characterization of CRP0770 Genes

Plant defensins and DEFLs are widespread in plants. We have studied here one group of DEFLs in Arabidopsis. The DEFL0770 group consists of seven genes in Arabidopsis. All seven genes encode cysteine-rich peptides with signal peptides, indicating secretion of the encoded peptides. This is generally known for plant defensins [22] although there has been a report of a plant defensin with signal peptide which is not secreted [36]. We have identified related genes only in species of the *Brassicaceae* family, which indicates a recent evolution of these genes. Nothing is known about these genes and the peptides they encode. Here we did not study the evolution or neofunctionalization of these DEFLs in detail. Our main goal was the functional characterization of one member of the DEFL0770 group, *At3g59930*.

### 3.2. Expression

Our RNAseq analysis found five DEFL0770 genes to be expressed in roots, four of them at a very high level. Information is also available from a study that used a microarray of all Arabidopsis DEFL genes [43]. That study also found strong expression of *At3g59930* and *At5g33355* in roots while expression of the other five genes in roots was low, which is partly in contrast to our RNAseq data. Strong expression was found in seedlings for the genes *At3g05727*, *At3g05730*, *At3g59930* and *At5g33355*, while expression in siliques was very low for all the genes. In the case of *At3g59930*, we did not find expression in seedlings using a promoter::GUS line. An explanation for the differences between our data and the results from the microarray analysis [43] might be that the probes that were used on the microarray had a rather high similarity. This is not surprising given the high similarity between the DEFL0770 genes. That was the reason that we had excluded some of the genes from a previous study with Affymetrix Genechips [40]. It might also be possible that the seedlings that were used for the microarray analysis still contained roots.

The DEFL microarray [43] was also used to study expression after infection with pathogens. Leaves were infected with *A. brassicicola*, leading to a strong induction of *At3g59930* and *At5g33355*. Contrary to that, our *At3g59930*::GUS line did not show any expression after inoculation with *A. brassicicola*. The reason for that is currently not clear; however, it might be possible that only certain strains of *A. brassicicola* induce expression of *At3g59930.* Similarly, in case of *P. syringae*, no induction of any DEFL0770 gene was found [43] and also our *At3g59930*::GUS line did not show any expression after inoculation with *P. syringae*.

A different situation was found concerning expression of the DEFL0770 genes after infection of the plants with the plant pathogenic nematode *H. schachtii*. The RNAseq data showed strong suppression of the DEFL0770 genes which was confirmed for *At3g59930* using GUS analysis. The suppression was already visible at 1 dpi and at later stages extended beyond syncytia. It is most likely that effectors produced by the nematode [11] are involved in suppression of the *At3g59930* gene and the other genes of the DEFL0770 group for which our RNAseq data have shown a downregulation.

### 3.3. Antimicrobial Activity and Effect of Overexpression

Induction of DEFL0770 genes in response to *A. brassicicola* and downregulation of expression in syncytia indicated an involvement of the genes in plant defense. It is known that many plant defensins and DEFL peptides have antimicrobial activity in vitro [45,46] Examples of DEFL peptides from plants include, for instance, peptides from *Phaseolus vulgaris* [47,48] *Trigonella foenum-graecum* seeds [49] and from *Gymnocladus chinensis* seeds [50]. We have recently shown that two plant defensin-related peptides, which we called plant defensin-like, have antimicrobial activity against bacteria and especially fungi (Omidvar and Bohlmann, unpublished results). There are also many DEFL peptides from various animal species that have antimicrobial activity in vitro. They include defensin-like peptides from a tick [51] and from a manila clam [52].

To test the antimicrobial activity of the At3g59930 peptide, we had to produce it in *E. coli*. We did not find antimicrobial activity against the bacteria that we tested. This may not be surprising, as the reported antimicrobial activity of defensins and DEFLs is mostly against fungi and less against bacteria. Antifungal activity was found against *B. cinerea* with an IC_50_ of approximately 50 µg/mL, while no activity was found against *F. oxysporum*.

Plant pathogenic sedentary biotrophic nematodes such as *H. schachtii* take up nutrients only from the feeding sites that they induce in plant roots. To test the effect of the At3g59930 peptide on *H. schachtii* we, therefore, produced transgenic lines that overexpressed the peptide. Tests with *H. schachtii* clearly showed that these lines were less susceptible to the nematodes. There was a decrease in the number of females but an increase in the number of males. This corresponded with a smaller size of females and of syncytia in overexpression lines compared to wild type. These data indicate that the overexpression of the At3g59930 peptide did not have a direct effect on the nematodes but rather an inhibitory effect of the development of the syncytia. Smaller syncytia would provide fewer nutrients to the nematodes, leading to smaller and fewer females, while the number of males that need fewer nutrients would increase. It has been known for some time that more male but fewer female *H. schachtii* nematodes develop under unfavourable conditions leading to fewer and perhaps smaller female nematodes in the next generation [53].

Our results have uncovered only weak antimicrobial activity of At3g59930. From the microorganisms tested, only *B. cinerea* was inhibited by this peptide at higher concentrations. This effect was not strong enough to increase the resistance of overexpression lines against *B. cinerea*. On the contrary, there was a weak increase of susceptibility for which we have no explanation at the moment. The only resistance increase of the overexpression lines was against *H. schachtii*. However, as outlined above, this higher resistance was probably not due to a direct effect of the peptide against the nematodes. This leaves the possibility of an indirect effect, perhaps on the development of the syncytia. It has been shown for plant defensins that they can be involved in different biological activities, e.g., signal transduction pathways [54], pollen activity [55], root hair formation [56] and antioxidant activities [57]. Further work will be needed to address these possible functions. There have also been several reports that plant defensins and DEFLs can bind heavy metals [25]. Some examples have already been mentioned in the introduction. In this regard it is interesting that *At3g59930* has been reported to be stronger expressed under conditions of zinc deficiency [58]. This might indicate that At3g59930 could bind zinc. However, in preliminary experiments, we could not demonstrate that the At3g59930 peptide binds zinc (data not shown).

## 4. Materials and Methods

### 4.1. Cloning of Vectors

The binary vector pMAA-Red [59] was used to produce an overexpression construct and a promoter::GUS fusion. The overexpression vector was cloned as follows: the *At3g59930* coding sequence was amplified from genomic Arabidopsis DNA by PCR using the primers gAt3g59930forNco and gAt3g59930revBam (Table S5) containing the restriction sites NcoI and BamHI, respectively. The PCR product was digested with NcoI and BamHI and ligated into the vector pMAA-Red digested with the same enzymes to replace the GUS sequence. The vector construct was confirmed by sequencing.

For cloning of the promoter::GUS fusion, the promoter region (1030 bp upstream of the start codon) was amplified by PCR with the primers pAt3g59930forEco and pAt3g59930revNco using as template Arabidopsis genomic DNA. The PCR introduced restriction sites for EcoRI and NcoI, respectively. The PCR fragment was digested with EcoRI and NcoI and ligated to the large vector fragment of pMAA-Red digested with the same enzymes to replace the 35S promoter with the *At3g59930* promoter. The construct was verified by sequencing.

An expression vector for *E. coli* was constructed from a modified pETtrx1a vector [60] having an NdeI site at the start codon and a BamHI site behind the stop codon. It contains a His-tag in front of the thioredoxin sequence. A TEV site was included to allow the separation of the fusion tag from the At3g59930 peptide. The thioredoxin part was amplified by PCR using primers pETtrxfor1 and pETtrxTEVrev. The coding sequence of At3g59930 without signal peptide and intron was produced as follows: The sequence was amplified in 2 parts using the primers TEV1-59930for and At3g59930Mrev for part 1 and At3g59930Mfor and gAt3g59930revBam for part 2. Both parts were then combined and amplified with primers TEV1-59930for and gAt3g59930revBam which eliminated the intron. Finally, the thioredoxin fusion fragment and the At3g59930 fragment were combined and amplified with primers pETtrxfor1 and gAt3g59930revBam. This final PCR fragment was digested with NdeI and BamHI and cloned into the modified pETtrx1a vector digested with the same enzymes. The final expression vector was verified by sequencing.

### 4.2. Plant Material and Growth Conditions

Arabidopsis (ecotype Columbia) seeds were produced by growing the plants on soil in a growth chamber at 25 °C in a 16 h light and 8 h dark cycle. For fungal infection assays, plants were grown on soil at 25 °C in an 8 h light and 16 h dark cycle. Arabidopsis seeds were surface sterilized for 20 min in 6% (*w*/*v*) sodium hypochlorite and subsequently washed three times with sterile water. Plants were grown under sterile conditions in vitro on either Murashige and Skoog (MS) medium or Knop medium [61].

### 4.3. Arabidopsis Transformation

Binary vectors were introduced into *Agrobacterium tumefaciens* GV3101 using the freeze-thaw method [62]. Arabidopsis plants were transformed by a modified floral dip method [63]. Transformed seeds were identified as described [59] and transferred to soil for seed production.

Ten independent transgenic plants of the promoter::GUS construct were generated and tested for GUS activity to choose a representative line. This was then grown further to produce homozygous seeds. For overexpression, we generated 15 independent transgenic T2 lines. These were tested for expression level of the transgene using RT-PCR (Figure S3) with the primers described in Table S5. The lines with the best expression level were then made homozygous for resistance tests.

### 4.4. RNA Isolation

Plant material was frozen in liquid nitrogen and ground to a fine powder using mortar and pestle and 80–100 mg was transferred into a sterile 1.5 mL Eppendorf tube. Total RNA was isolated by using the Qiagen RNeasy mini kit (QIAGEN, Hilden, Germany) including the Qiagen RNase-free DNase kit. All steps were performed according to manufacturer’s instructions. RNA purity and quantity was measured using the NanodropTM 2000C supported with the NanodropTM software. RNA samples were stored at −80 °C.

### 4.5. Reverse Transcriptase PCR (RT-PCR)

First strand cDNA was synthesized from 5 µg RNA per sample by using peqGOLD M-MuLV H plus reverse transcriptase (PeqLab Biotechnologie GmbH, Erlangen, Germany) and the gene specific primer At3g59930Rtrev (Table S5) according to manufacturer’s protocol. The reaction contained 4 µL RNA template, 8 µL RNase free water, 1 µL primer, 4 µL 5x reverse transcriptase reaction buffer, 2 µL dNTPs (10 mM) and 1 µL reverse transcriptase (PeqLab Biotechnologie GmbH, Erlangen, Germany). After incubation at 42 °C for 60 min, the reaction was terminated by heating at 70 °C for 10 min. The cDNA product was stored at −20 °C. PCR was done using primers At3g59930Rtfor and At3g59930Rtrev (Table S5).

### 4.6. RNAseq

For RNAseq analysis 10 μL of RNA of each sample were sent to GATC Eurofins (Konstanz, Germany). Samples were analysed with Genome Sequencer Illumina HiSeq in mode HiSeq 4000 50 bp SR. The raw data of mapped reads were normalized calculating transcripts per million (TPM). The counts of each transcript were divided by its length. All normalized transcripts were summed up and divided by 1,000,000, creating the scaling factor. Every single transcript is divided by the scaling factor resulting in a TPM value.

### 4.7. GUS Reporter Analysis

We used a histochemical detection of GUS activity according to [64]. The GUS solution contained X-gluc (Biomol, Hamburg, Germany) in 0.1 M sodium phosphate buffer pH 7.0, 0.1% Triton-X 100, 0.5 mM K_3_[Fe(CN)_6_], 0.5 mM K_4_[Fe(CN)_6_] and 10 mM Na_2_EDTA. Different plant parts and growth stages were incubated in GUS solution at 37 °C overnight. After staining, probes were incubated in 70% (*v*/*v*) ethanol to remove chlorophyll from photosynthetic tissues. Stained plants were photographed. Similarly, for GUS staining of nematode infection sites, the infected roots of promoter::GUS plants at different time points after inoculation were incubated with X-gluc at 37 °C and destained with 70% (*v*/*v*) ethanol. Infected as well as uninfected roots were photographed with an inverse microscope (Axiovert 200M; Zeiss, Hallerbergmoos, Germany) having an integrated camera (AxioCam MRc5; Zeiss).

### 4.8. Nematode Resistance Tests

*H. schachtii* cysts were harvested from in vitro stock cultures kept on mustard (*Sinapis alba* cv. Albatros) roots on 0.2% Knop medium supplemented with 2% sucrose [61]. Hatching of J2 larvae from the cysts was stimulated by soaking the cysts in 3 mM ZnCl_2_ for 5–6 days. Larvae were collected in 20 µm sieves and subsequently surface sterilized by soaking in 0.05% HgCl_2_ for 2–3 min followed by three times washing in sterile water. Finally, J2 larvae were resuspended in 0.5% (*w*/*v*) gelrite (Duchefa, Haarlem, The Netherlands) and mixed thoroughly.

Roots of 12-day old Arabidopsis seedlings were inoculated with approximately 50–60 freshly hatched J2 larvae per plant under sterile conditions. The number of male and female nematodes per plant was counted at 12 dpi. On the following day, pictures of female syncytia and female nematodes (longitudinal optical sections) were taken using an inverse microscope (Axiovert 200M; Zeiss AG, Germany). These pictures were used to determine the average size of female nematodes and associated syncytia. For this, the Axiovision kontour software (Zeiss AG, Germany) was used. All experiments were repeated independently at least three times. Each experiment contained 18–20 plants per line with 2 plants per Petri dish.

The data regarding number of nematodes and sizes of nematodes and syncytia were analyzed using single factor ANOVA (*p* < 0.05). As the F-statistic was greater than F-critical, a least significance test (LSD) was applied.

### 4.9. Fungal Infection Assays

*B. cinerea* (MG 1401) was a gift from Dragana Bandia and Dr. Joseph Strauss (Austrian Institute of Technology, Seibersdorf, Austria). For infection assays, *B. cinerea* was maintained for 2 weeks at room temperature on malt extract agar. Spores were collected and resuspended in 1/2 potato dextrose broth and adjusted at a density of 2.5 × 10^5^ spores/mL. Then, leaves of 4 weeks old plants were challenged with one 5 μL droplet of the spore suspension. Inoculated plants were kept under transparent plastic cover to maintain high humidity. The necrotic lesions were photographed and measured at 3 dpi. Three independent experiments were conducted, and each experiment included approximately 20 replicates.

### 4.10. Expression and Purification of At3g59930 Peptide

The recombinant pETtrx::At3g59930 vector was transformed into the *E.coli* strain C3030 Shuffle [65]. A 30 mL LB medium pre-culture (containing 100 µg/mL kanamycin) was inoculated with a single colony. After overnight incubation at 37 °C and 140 rpm, 12.5 mL of the pre-culture was used to inoculate 500 mL of LB medium (containing 100 µg/mL kanamycin). This culture was grown at 30 °C and 140 rpm to an OD_600_ of 0.6. Afterwards, the expression was induced by addition of 0.75 mM IPTG. The induced culture was incubated overnight at 16 °C and 140 rpm. The cells were harvested by centrifugation for 30 min at 4 °C (Sorval RC6+ centrifuge, Thermo Scientific, Vienna, Austria). Cells were resuspended in 30 mL of ice-cold buffer (20 mM NaH_2_PO_4_, 0.5 M NaCl, 20 mM imidazole, pH 7.4). After lysis in an ice-water bath by sonication (Sonifier W-250D, Branson Ultrasonics, Dietzenbach, Germany) the cell lysate was clarified by centrifugation at 17,000 rpm for 30 min at 4 °C.

For purification of the tagged fusion protein, immobilized metal ion affinity chromatography was used. Briefly, the supernatant after the second centrifugation was collected and incubated with Ni-NTA agarose beads resin (QIAGEN) equilibrated with binding buffer (20 mM NaH_2_PO_4_, 0.5 M NaCl, 20 mM Imidazole, pH 7.4) for 3–4 h with slow shaking at 4 °C. Afterwards, the suspension was loaded to an empty chromatography column (CHROMABOND^®^) and the flow-through was collected. The column was washed with 5 CV binding buffer (20 mM NaH_2_PO_4_, 0.5 M NaCl, 20 mM Imidazole, pH 7.4) followed by 5 CV of elution buffer (20 mM NaH_2_PO_4_, 0.5 M NaCl, 500 mM Imidazole, pH 7.4). Flow-through and the eluted fractions were analyzed by SDS-PAGE [66] on a Mini PROTEAN Tetra Cell 1mm (BioRAD, Vienna, Austria). The Thermo Scientific™ Pierce™ BCA Protein Assay kit was used for protein quantification. The fusion protein was cleaved using TEV protease and cleavage was confirmed by PAGE. Finally, the At3g59930 peptide was purified by reverse phase chromatography on an Äkta purifier10 system (GE Healthcare, Chicago, IL, USA) equipped with a 1ml HisTrap FF column. The purified peptide was run on a PAGE gel and silver stained according to [67].

### 4.11. Antimicrobial Assays

Antifungal assays were done according to [68] in clear 96 well cell culture microplates (PS, TC, F-bottom with a lid, Greiner Bio-One, Frickenhausen, Germany). We used 2 different fungi, *B. cinerea* and *F. oxysporum* f.sp. *matthiolae* which were grown on PDA plates for two weeks at room temperature. *B. cinerea* was grown in the dark and *F. oxysporum* in the light on the lab bench. The plates were flooded with 10 mL of sterile water to harvest the spores. The spore suspension was filtered through 2 layers of cheesecloth. Spores were counted with a Thoma hemocytometer and the spore concentration was adjusted to 2 × 10^4^ spores/mL in ½ concentrated PDB. For the assay, 25 µL of the test peptide were combined with 75 µL of the spore suspension. Final peptide concentrations were 100 µg/mL, 50 µg/mL, 25 µg/mL, 12.5 µg/mL, 6.25 µg/mL, 3.125 µg/mL, 1.56 µg/mL and 0 µg/mL. Nystatin was used as a control. The OD_600_ was measured on a Fluostar Omega Plate reader (BMG Labtech, Kumberg, Austria) using the well scan setting with a diameter of 3 mm and 9 measuring points and double orbital shaking for 30 s. before the measurement. OD was measured at the beginning of the experiment and again after 24 h and 48 h incubation at room temperature in the dark. To calculate percentage of growth inhibition, the median of all measuring points per well was used as follows:

% growth inhibition = 100 × (absorbance of control − absorbance of test)/absorbance of control

Bacteria were tested using the resazurin microplate assay as described by [69] with some modifications. Test organisms were *A. tumefaciens* GV3101 and *P. syringae* pv. tomato DC3000. Bacteria were grown in 5 mL LB medium over night at 30 °C. The cultures were then centrifuged for 10 min at 7000 rpm. Bacteria were resuspended in 1% tryptone and diluted to an OD_600_ of 0.05 using again 1% tryptone. The resazurin assay was carried out in a 96 well microtiter plate. In each row, two-fold serial dilutions of the At3g59930 peptide in 50 μL of minimal medium were prepared. The range of concentrations was 100–0.78 µg/mL. Then, 48 µL of bacterial inoculum (adjusted to OD_600_ of 0.05) followed by 2 µL resazurin were added to each well. A row of wells containing kanamycin in the same concentration as the tested peptide was included. In addition, growth control and sterility control with no At3g59930 peptide were also added to each row. Finally, the plates were covered, sealed with parafilm and incubated 24–48 h at the optimum conditions for the tested pathogen. Color change was then observed visually. In this regard, changes of color from blue to pink indicate the inability of the peptide to inhibit the growth of the microbial pathogen and therefore was considered negative, while the wells that showed no changes in color (remain blue) were recorded as positive. The lowest concentration with no color changes was considered the minimum inhibitory value (MIC) for the tested pathogen. Each experiment was done in triplicate and repeated three times.

### 4.12. In Silico Analysis of CRP0770 Genes

The peptide, complementary DNA sequences (CDS), and genomic sequences of the seven AtDEFLs were retrieved from Phytozome [70]. Peptide sequences were aligned in MEGA software v. 7.0 [71] by using the built-in MUSCLE tool [72]. ModelFinder was used for selection of the appropriate model of substitution [73]. A tree was constructed in iQ-TREE [74] using ultrafast bootstrap [75] with 1000 replicates. Physical chromosomal mapping of the genes was carried out in MapChar [76]. Gene structures were visualized in GSDS 2.0 [77]. SignalP (http://www.cbs.dtu.dk/services/SignalP/, accessed on 7 October 2021) was used to predict signal peptides [78]. Conserved motif analysis was done in The MEME Suite [79]. The figure panels were generated by exporting the figures from respective software and merged in Microsoft Power Point^®^ Office 2013.

The homologs of the selected AtDEFLs were searched by BLASTP against proteomes of all the sequenced plants (85 plant species) available on Phytozome v.12.1 with expect threshold E-value = −20 and the peptide sequences were downloaded. The sequence alignment and ML tree construction was done as reported above.

### 4.13. Statistical Analysis

Data regarding the number of nematodes, the size of female nematodes and syncytia and lesion diameter were analysed using single factor ANOVA (*p* < 0.05), while the mean comparisons were performed using a least significant difference (LSD) test at a 95% level of confidence.

## 5. Conclusions

We have shown that the DEFL0770 gene *At3g59930*, which is only expressed in roots, is strongly downregulated after infection with *H. schachtii*. Overexpression lines are more resistant to *H. schachtii* infection. Our data indicate that At3g59930 is involved in resistance to nematodes.

## Data Availability

Data is contained within the article or supplementary material.

## Supplementary Materials

The following supporting information can be downloaded at: https://www.mdpi.com/article/10.3390/plants11030280/s1, Figure S1: Leaves of the *At3g59930* promoter::GUS line were stained for GUS at different time points after infection with A. *A. brassicicola*; B. *B. cinerea*; C. *P. syringae*, Figure S2: Diagram of the vector for expression of *At3g59930* in *E. coli*, Figure S3: RT-PCR of overexpression lines of *At3g59930*, Table S1: Expression of DEFL0770 genes in Arabidopsis control roots and 15-days old syncytia induced by H. schachtii, Table S2: Microarray data from Tesfaye et al. (2013), Table S3: Microarray data from Tesfaye et al. (2013), Table S4: Microarray data from Tesfaye et al. (2013), Table S5: Primers used in this work.

## Abbreviations

AMP	antimicrobial peptide
CRP	cysteine-rich peptide
DEFL	defensin-like peptide
dpi	days post inoculation
IPTG	Isopropyl-β-D-thiogalactopyranosid
CV	column volume
TEV	tobacco etch virus
PAGE	polyacrylamide gel electrophoresis

## References

[1] Bohlmann H. (2015). Introductory chapter on the basic biology of cyst nematodes. Advances in Botanical Research.

[2] Jones M.G.K., Northcote D.H. (1972). Nematode-induced syncytium-A multinucleate transfer cell. J. Cell Sci..

[3] Sobczak M., Golinowski W. (2011). The Structure of Syncytia. Genomics and Molecular Genetics of Plant-Nematode Interaction.

[4] Ali M.A., Azeem F., Li H., Bohlmann H. (2017). Smart parasitic nematodes use multifaceted strategies to parasitize plants. Front. Plant Sci..

[5] Wieczorek K., Golecki B., Gerdes L., Heinen P., Szakasits D., Durachko D.M., Cosgrove D.J., Kreil D.P., Puzio P.S., Bohlmann H. (2006). Expansins are involved in the formation of nematode-induced syncytia in roots of Arabidopsis thaliana. Plant J..

[6] Bohlmann H., Sobczak M. (2014). The plant cell wall in the feeding sites of cyst nematodes. Front. Plant Sci..

[7] Siddique S., Sobczak M., Tenhaken R., Grundler F.M.W., Bohlmann H. (2012). Cell wall ingrowths in nematode induced syncytia require UGD2 and UGD3. PLoS ONE.

[8] Sobczak M., Golinowski W., Grundler F.M. (1997). Changes in the structure of Arabidopsis thaliana roots induced during development of males of the plant parasitic nematode Heterodera schachtii. Eur. J. Plant Pathol..

[9] Niebel A., de Almeida Engler J., Hemerly A., Ferreira P., Inzé D., Van Montagu M., Gheysen G. (1996). Induction of cdc2a and cyc1At expression in Arabidopsis thaliana during early phases of nematode-induced feeding cell formation. Plant J..

[10] Jaubert S., Ledger T.N., Laffaire J.B., Piotte C., Abad P., Rosso M.N. (2002). Direct identification of stylet secreted proteins from root-knot nematodes by a proteomic approach. Mol. Biochem. Parasitol..

[11] Hewezi T., Baum T.J. (2013). Manipulation of plant cells by cyst and root-knot nematode effectors. Mol. Plant-Microbe Interact..

[12] Gilchrist D.G. (1998). Programmed cell death in plant disease: The purpose and promise of cellular suicide. Annu. Rev. Phytopathol..

[13] Piffanelli P., Devoto A., Schulze-Lefert P. (1999). Defence signalling pathways in cereals. Curr. Opin. Plant Biol..

[14] Van Loon L.C., Rep M., Pieterse C.M.J. (2006). Significance of inducible defense-related proteins in infected plants. Annu. Rev. Phytopathol..

[15] Bohlmann H., Apel K. (1991). Thionins. Annu. Rev. Plant Biol..

[16] Broekaert W.F., Terras F., Cammue B., Osborn R.W. (1995). Plant Defensins: Novel Antimicrobial Peptides as Components of the Host Defense System. Plant Physiol..

[17] Garcia-Olmedo F., Molina A., Segura A., Moreno M. (1995). The defensive role of nonspecific lipid-transfer proteins in plants. Trends Microbiol..

[18] Cammue B.P., De Bolle M.F., Terras F.R., Proost P., Van Damme J., Rees S.B., Vanderleyden J., Broekaert W.F. (1992). Isolation and Characterization of a Novel Class of Plant Antimicrobial Peptides from Mirabilis jalapa L. seeds. J. Biol. Chem..

[19] Nielsen K.K., Nielsen J.E., Madrid S.M., Mikkelsen J.D. (1997). Characterization of a new antifungal chitin-binding peptide from sugar beet leaves. Plant Physiol..

[20] Craik D.J. (2012). Host-defense activities of cyclotides. Toxins.

[21] Segura A., Moreno M., Madueño F., Molina A., García-olmedo F. (1999). Snakin-1, a Peptide from Potato That Is Active Against Plant Pathogens. Mol. Plant-Microbe Interact..

[22] Tam J.P., Wang S., Wong K.H., Tan W.L. (2015). Antimicrobial Peptides from Plants. Pharmaceuticals.

[23] Sathoff A.E., Velivelli S., Shah D.M., Samac D.A. (2019). Plant defensin peptides have antifungal and antibacterial activity against human and plant pathogens. Phytopathology.

[24] Chen K.-C., Lin C.-Y., Kuan C.-C., Sung H.-Y., Chen C.-S. (2003). A Novel Defensin Encoded by a Mungbean cDNA Exhibits Insecticidal Activity against Bruchid. J. Agric. Food Chem..

[25] Mirouze M., Sels J., Richard O., Czernic P., Loubet S., Jacquier A., François I.E.J.A., Cammue B.P.A., Lebrun M., Berthomieu P. (2006). A putative novel role for plant defensins: A defensin from the zinc hyper-accumulating plant, Arabidopsis halleri, confers zinc tolerance. Plant J..

[26] Bloch C., Richardson M. (1991). A new family of small (5 kDa) protein inhibitors of insect α-amylases from seeds or sorghum (Sorghum bicolor (L) Moench) have sequence homologies with wheat γ-purothionins. FEBS Lett..

[27] Melo F.R., Rigden D.J., Franco O.L., Mello L.V., Ary M.B., Grossi-De-Sa M.F., Bloch C. (2002). Inhibition of trypsin by cowpea thionin: Characterization, molecular modeling, and docking. Proteins Struct. Funct. Genet..

[28] Coninck B.D.E., Cammue B.P.A., Thevissen K. (2013). Modes of antifungal action and in planta functions of plant defensins and defensin-like peptides. Fungal Biol. Rev..

[29] Li Z., Zhou M., Zhang Z., Ren L., Du L., Zhang B., Xu H., Xin Z. (2011). Expression of a radish defensin in transgenic wheat confers increased resistance to Fusarium graminearum and Rhizoctonia cerealis. Funct. Integr. Genom..

[30] Thomma B.P.H.J., Cammue B.P.A., Thevissen K. (2002). Plant defensins. Planta.

[31] Thomma B.P.H.J., Eggermont K., Penninckx I.A.M.A., Mauch-Mani B., Vogelsang R., Cammue B.P.A., Broekaert W.F. (1998). Separate jasmonate-dependent and salicylate-dependent defense-response pathways in arabidopsis are essential for resistance to distinct microbial pathogens. Proc. Natl. Acad. Sci. USA.

[32] Penninckx I.A.M.A., Thomma B.P.H.J., Buchala A., Métraux J.P., Broekaert W.F. (1998). Concomitant activation of jasmonate and ethylene response pathways is required for induction of a plant defensin gene in Arabidopsis. Plant Cell.

[33] De Coninck B.M.A., Sels J., Venmans E., Thys W., Goderis I.J.W.M., Carron D., Delauré S.L., Cammue B.P.A., De Bolle M.F.C., Mathys J. (2010). Arabidopsis thaliana plant defensin AtPDF1.1 is involved in the plant response to biotic stress. New Phytol..

[34] Hsiao P.Y., Cheng C.P., Koh K.W., Chan M.T. (2017). The Arabidopsis defensin gene, AtPDF1.1, mediates defence against Pectobacterium carotovorum subsp. carotovorum via an iron-withholding defence system. Sci. Rep..

[35] Luo J.S., Yang Y., Gu T., Wu Z., Zhang Z. (2019). The Arabidopsis defensin gene AtPDF2.5 mediates cadmium tolerance and accumulation. Plant Cell Environ..

[36] Luo J.S., Gu T., Yang Y., Zhang Z. (2019). A non-secreted plant defensin AtPDF2.6 conferred cadmium tolerance via its chelation in Arabidopsis. Plant Mol. Biol..

[37] Silverstein K.A.T., Graham M.A., Paape T.D., Vandenbosch K.A. (2005). Genome Organization of More Than 300 Defensin-Like Genes in Arabidopsis 1 [w]. Plant Physiol..

[38] Takeuchi H., Higashiyama T. (2012). A Species-Specific Cluster of Defensin-Like Genes Encodes Diffusible Pollen Tube Attractants in Arabidopsis. PLoS Biol..

[39] Amien S., Kliwer I., Márton M.L., Debener T., Geiger D., Becker D., Dresselhaus T. (2010). Defensin-like ZmES4 mediates pollen tube burst in maize via opening of the potassium channel KZM1. PLoS Biol..

[40] Szakasits D., Heinen P., Wieczorek K., Hofmann J., Wagner F., Kreil D.P., Sykacek P., Grundler F.M.W., Bohlmann H. (2009). The transcriptome of syncytia induced by the cyst nematode Heterodera schachtii in Arabidopsis roots. Plant J..

[41] Wu J., Jin X., Zhao Y., Dong Q., Jiang H., Ma Q. (2016). Evolution of the defensin-like gene family in grass genomes. J. Genet..

[42] Murat F., Louis A., Maumus F., Armero A., Cooke R., Quesneville H., Crollius H.R., Salse J. (2015). Understanding Brassicaceae evolution through ancestral genome reconstruction. Genome Biol..

[43] Tesfaye M., Silverstein K.A.T., Nallu S., Wang L., Botanga C.J., Gomez S.K., Costa L.M., Harrison M.J., Samac D.A., Glazebrook J. (2013). Spatio-Temporal Expression Patterns of Arabidopsis thaliana and Medicago truncatula Defensin-Like Genes. PLoS ONE.

[44] Bruggeman M., Ijakipour H., Stamboulis A. (2019). Defensin-Like Peptides and Their Antimicrobial Activity in Free-Form and Immobilized on Material Surfaces. Peptide Synthesis.

[45] Parisi K., Shafee T.M.A., Quimbar P., van der Weerden N.L., Bleackley M.R., Anderson M.A. (2019). The evolution, function and mechanisms of action for plant defensins. Semin. Cell Dev. Biol..

[46] Sathoff A.E., Samac D.A. (2019). Antibacterial activity of plant defensins. Mol. Plant-Microbe Interact..

[47] Wong J.H., Ip D.C.W., Ng T.B., Chan Y.S., Fang F., Pan W.L. (2012). A defensin-like peptide from Phaseolus vulgaris cv. “King Pole Bean”. Food Chem.

[48] Wu X., Sun J., Zhang G., Wang H., Ng T.B. (2011). An antifungal defensin from Phaseolus vulgaris cv. “Cloud Bean”. Phytomedicine.

[49] Oddepally R., Guruprasad L. (2015). Isolation, purification, and characterization of a stable defensin-like antifungal peptide from Trigonella foenum-graecum (fenugreek) seeds. Biochemistry.

[50] Wong J.H., Ng T.B. (2003). Gymnin, a potent defensin-like antifungal peptide from the Yunnan bean (Gymnocladus chinensis Baill). Peptides.

[51] Li F., Gao Z., Wang K., Zhao Y., Wang H., Zhao M., Zhao Y., Bai L., Yu Z., Yang X. (2021). A novel defensin-like peptide contributing to antimicrobial and antioxidant capacity of the tick Dermacentor silvarum (Acari: Ixodidae). Exp. Appl. Acarol..

[52] Yang D., Zhang Q., Wang Q., Chen L., Liu Y., Cong M., Wu H., Li F., Ji C., Zhao J. (2018). A defensin-like antimicrobial peptide from the manila clam Ruditapes philippinarum: Investigation of the antibacterial activities and mode of action. Fish Shellfish Immunol..

[53] Grundler F.B.M., Wyss U. (1991). Influence of Changes in the Nurse Cell System (Syncytium) on Sex Determination and Development of the Cyst Nematode Heterodera schachtii: Total Amounts of Proteins and Amino Acids. Phytopathology.

[54] Lay F., Anderson M. (2005). Defensins—Components of the Innate Immune System in Plants. Curr. Protein Pept. Sci..

[55] Stotz H.U., Thomson J.G., Wang Y. (2009). Plant defensins: Defense, development and application. Plant Signal. Behav..

[56] Allen A., Snyder A.K., Preuss M., Nielsen E.E., Shah D.M., Smith T.J. (2008). Plant defensins and virally encoded fungal toxin KP4 inhibit plant root growth. Planta.

[57] Huang G.J., Deng J.S., Chen H.J., Huang S.S., Liao J.C., Hou W.C., Lin Y.H. (2012). Defensin protein from sweet potato (*Ipomoea batatas* [L.] Lam ’Tainong 57’) storage roots exhibits antioxidant activities in vitro and ex vivo. Food Chem..

[58] Van De Mortel J.E., Villanueva L.A., Schat H., Kwekkeboom J., Coughlan S., Moerland P.D., Van Themaat E.V.L., Koornneef M., Aarts M.G.M. (2006). Large expression differences in genes for iron and zinc homeostasis, stress response, and lignin biosynthesis distinguish roots of Arabidopsis thaliana and the related metal hyperaccumulator Thlaspi caerulescens. Plant Physiol..

[59] Ali M.A., Shah K.H., Bohlmann H. (2012). pMAA-Red: A new pPZP-derived vector for fast visual screening of transgenic Arabidopsis plants at the seed stage. BMC Biotechnol..

[60] Bogomolovas J., Simon B., Sattler M., Stier G. (2009). Screening of fusion partners for high yield expression and purification of bioactive viscotoxins. Protein Expr. Purif..

[61] Sijmons P.C., Grundler F.M., von Mende N., Burrows P.R., Wyss U. (1991). Arabidopsis thaliana as a new model host for plant-parasitic nematodes. Plant J..

[62] Holsters M., de Waele D., Depicker A., Messens E., van Montagu M., Schell J. (1978). Transfection and transformation of Agrobacterium tumefaciens. MGG Mol. Gen. Genet..

[63] Logemann E., Birkenbihl R.P., Ülker B., Somssich I.E. (2006). An improved method for preparing Agrobacterium cells that simplifies the Arabidopsis transformation protocol. Plant Methods.

[64] Jefferson R.A. (1989). The GUS reporter gene system. Nature.

[65] Lobstein J., Emrich C.A., Jeans C., Faulkner M., Riggs P., Berkmen M. (2016). SHuffle, a novel Escherichia coli protein expression strain capable of correctly folding disulfide bonded proteins in its cytoplasm. Microb. Cell Fact..

[66] Schägger H. (2006). Tricine-SDS-PAGE. Nat. Protoc..

[67] Gharahdaghi F., Weinberg C.R., Meagher D.A., Imai B.S., Mische S.M. (1999). Mass spectrometric identification of proteins from silver-stained polyacrylamide gel: A method for the removal of silver ions to enhance sensitivity. Electrophoresis.

[68] Broekaert W.F., Terras F.R.G., Cammue B.P.A., Vanderleyden J. (1990). An automated quantitative assay for fungal growth inhibition. FEMS Microbiol. Lett..

[69] Sarker S.D., Nahar L., Kumarasamy Y. (2007). Microtitre plate-based antibacterial assay incorporating resazurin as an indicator of cell growth, and its application in the in vitro antibacterial screening of phytochemicals. Methods.

[70] Goodstein D.M., Shu S., Howson R., Neupane R., Hayes R.D., Fazo J., Mitros T., Dirks W., Hellsten U., Putnam N. (2012). Phytozome: A comparative platform for green plant genomics. Nucleic Acids Res..

[71] Kumar S., Stecher G., Li M., Knyaz C., Tamura K. (2018). MEGA X: Molecular evolutionary genetics analysis across computing platforms. Mol. Biol. Evol..

[72] Edgar R.C. (2004). MUSCLE: A multiple sequence alignment method with reduced time and space complexity. BMC Bioinform..

[73] Kalyaanamoorthy S., Minh B.Q., Wong T.K.F., Von Haeseler A., Jermiin L.S. (2017). ModelFinder: Fast model selection for accurate phylogenetic estimates. Nat. Methods.

[74] Nguyen L.T., Schmidt H.A., Von Haeseler A., Minh B.Q. (2015). IQ-TREE: A fast and effective stochastic algorithm for estimating maximum-likelihood phylogenies. Mol. Biol. Evol..

[75] Hoang D.T., Chernomor O., Von Haeseler A., Minh B.Q., Vinh L.S. (2017). UFBoot2: Improving the ultrafast bootstrap approximation. Mol. Biol. Evol..

[76] Voorrips R.E. (2002). Computer Note MapChart: Software for the Graphical Presentation of Linkage Maps and QTLs. J. Hered..

[77] Hu B., Jin J., Guo A., Zhang H., Luo J. (2015). Genome analysis GSDS 2.0: An upgraded gene feature visualization server. Bioinformatics.

[78] Almagro Armenteros J.J., Tsirigos K.D., Sønderby C.K., Petersen T.N., Winther O., Brunak S., von Heijne G., Nielsen H. (2019). SignalP 5.0 improves signal peptide predictions using deep neural networks. Nat. Biotechnol..

[79] Bailey T.L., Johnson J., Grant C.E., Noble W.S. (2015). The MEME Suite. Nucleic Acids Res..

