# Supplementary material for: Characterization of an Arabidopsis Defensin-like Gene Conferring Resistance against Nematodes
Source: Plants (Basel). 2022 Jan 21;11(3):280. doi: 10.3390/plants11030280 (PMC8838067; doi:10.3390/plants11030280)
Supplement: Supplementary file 1 [file plants-11-00280-s001.zip › plants-1546235-supplementary.pdf]

Supplementary materials

Table S1. Expression of DEFL0770 genes in Arabidopsis control roots and 15-days old syncytia induced by *H. schachtii*

gene_id	Root1	Root2	Root3	Syn1	Syn2	Syn3
At1g34047.2	7128.08	9318.98	10004	562.15	427.03	588.31
At2g36255.1	4954.69	4090.02	5510.27	441.9	370.69	425.22
At3g05727.1	1.57	0.74	0	0	0	0
At3g05730.1	0.33	0.39	1.94	0.04	0	0
At3g59930.1	6023.75	6383.24	7557.54	1290.3	815.74	1027.37
At4g11393.1	879.58	794.53	1205.11	89.4	53.84	87.11
At5g33355.1	8602.99	8329.14	11966.7	503.19	449.53	541.69

Shown are RNAseq data as transcripts per million (TPM) for control root segments (three independent experiments) and syncytia cut out from roots (three independent experiments)..

Table S2. Microarray data from Tesfaye et al. (2013)

Gene	INF	Root	sedl14	sedl7	siliques
At1g34047	16,8	34,1	17,9	33,4	24,7
At2g36255	10,6	11,4	11,0	10,5	17,4
At3g05727	21,3	55,4	3.091,3	1.105,9	47,0
At3g05730	21,3	72,5	4.439,3	5.577,3	15,5
At3g59930	24,3	805,8	448,9	1.405,3	40,8
At4g11393	12,5	14,3	17,4	17,8	17,9
At5g33355	20,1	385,6	221,9	1.138,6	18,5

Data show expression in different plant parts. INF, inflorescence; Root, root at 21d; sedl14, seedling at 14d; sedl7, seedling at 7d; siliques, siliques.

Table S3. Microarray data from Tesfaye et al. (2013)

Gene	ALTMOCK	ALTCO24	ALTDDE224
At1g34047	23,9	23,5	22,5
At2g36255	10,4	11,1	10,6
At3g05727	27,4	22,4	19,8
At3g05730	3.792,3	1.997,0	2.081,7
At3g59930	69,1	3.000,0	238,8
At4g11393	17,3	12,6	13,5
At5g33355	46,4	3.278,0	106,5

Data show expression in leaves after infection with *Alternaria brassicicola*: ALTMOCK, mock-inoculated Columbia leaf - control for *Alternaria*; ALTCO24, *Alternaria*_inoculated Columbia; ALTDDE224, *Alternaria* inoculated mutant dde2;

Table S4. Microarray data from Tesfaye et al. (2013)

Gene	PST3MOCK	PST9MOCK	AVR3	AVR9	DC3003	DC3009	Hrcc3	Hrcc9
At1g34047	16,5	15,8	18,0	15,9	15,5	16,0	15,9	15,8
At2g36255	11,3	11,1	9,6	8,9	10,0	8,4	9,5	9,1
At3g05727	17,3	19,3	16,9	18,8	16,8	18,2	15,8	17,3
At3g05730	275,5	566,3	71,9	247,6	66,4	349,3	114,0	349,1
At3g59930	12,3	28,6	17,0	32,4	13,1	90,8	12,9	37,7
At4g11393	13,8	12,4	12,8	12,1	12,1	10,4	13,3	14,2
At5g33355	15,8	23,8	14,2	23,5	15,0	51,1	17,5	33,5

Data show expression in leaves after infection with *Pseudomonas syringae* pv *tomato*: PST3MOCK, mock-inoculated control leaf for *Pseudomonas* at 3 h; PST9MOCK, mock-inoculated control leaf for *Pseudomonas* at 9 h; AVR3, AvrRpt2-inoculated leaf at 3h; AVR9, AvrRpt2-inoculated leaf at 9h; DC3003, DC3000-inoculated leaf at 3h; DC3009, DC3000-inoculated leaf at 9h; Hrcc3, Hrcc-inoculated leaf at 3h; Hrcc9, Hrcc-inoculated leaf at 9h (PtoDC3000hrcC is a strain unable to deliver effectors which induce PTI. PtoDC3000AvrRpt2 delivers AvrRpt2, an effector that triggers ETI in Col-0 strains compared to plants inoculated with wild type PtoDC3000.)

Table S5. Primers used in this work

Primer name	Sequence	Use
**gAt3g59930forNco**	AAGCCATGGCAAAGAACCTCAACTCC	Amplification of *At3g59930* coding sequence from genomic DNA
**gAt3g59930revBam**	TTAGGATCCTTACGATTTGTAGCAATGGCAG	
**pAt3g59930forEco**	GATGAATTCCAGTTTTACCTTCTTCC	Amplification of *At3g59930* promoter sequence
**pAt3g59930revNco**	TTGCCATGGCTTTAATGCTTTGCGTTTAG	
**At3g59930RTfor**	GGCAAAGAACCTCAACTCCG	RT-PCR for *At3g59930*
**At3g59930RTrev**	AGAGAGCTACACAACACGCT	
**At3g59930Mfor**	GTGGCTTCCACCGAAATCCTGAAGAG	Remove intron from *At3g59930*
**At3g59930Mrev**	CTCTTCAGGATTTCGGTGGAAGCCAC	
**pETtrxfor1**	GTCCGGCGTAGAGGATCG	Amplification of TRX fusion part ofvector pETtrx1a
**pETtrxTEVrev**	CTGAAAATAAAGATTCTCAGA	
**TEV1-59930for**	GAGAATCTTTATTTTCAGGCATGCTTCACGTTC	Combine *At3g59930* coding sequencewith TEV sequence of pETtrx1a

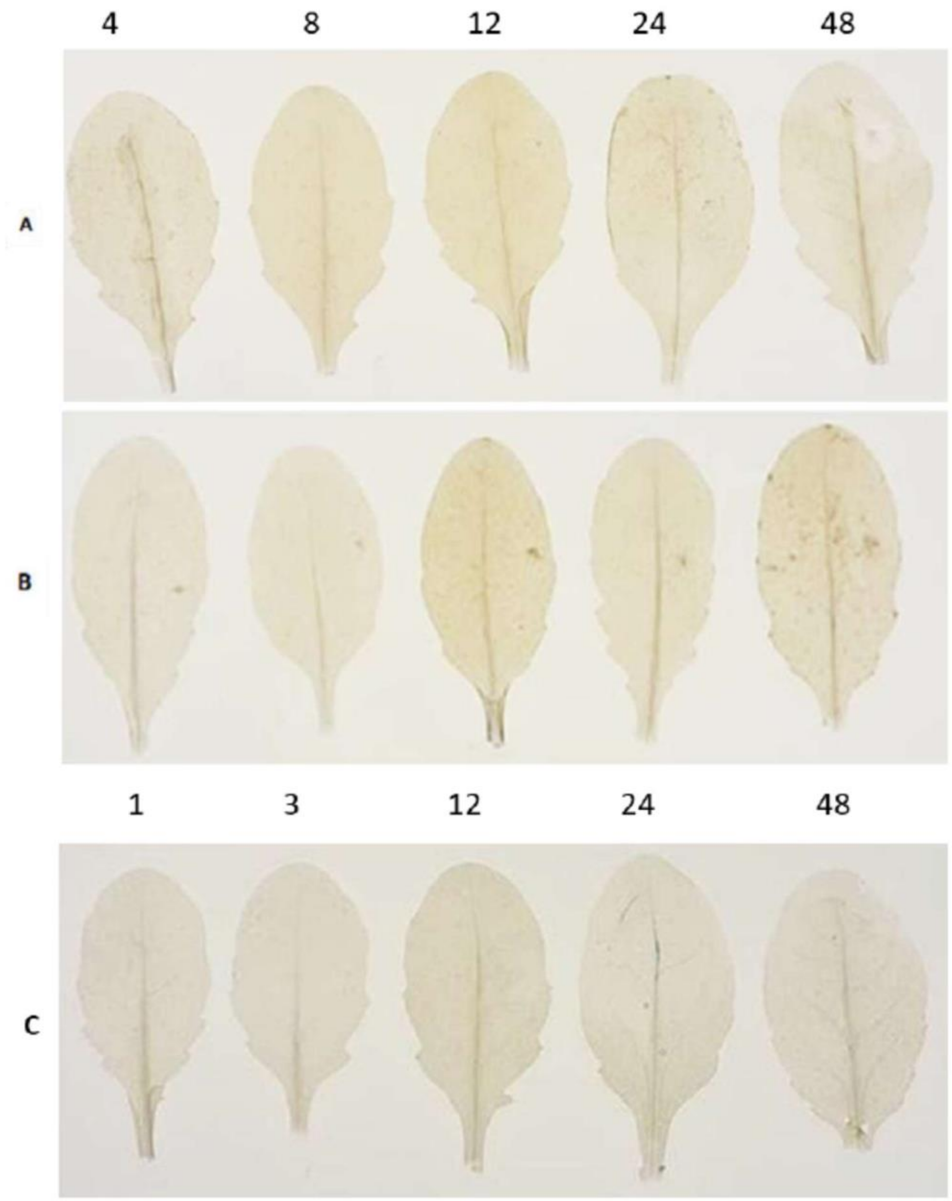

Figure S1. Leaves of the At3g59930 promoter::GUS line were stained for GUS atdifferent time points after infection with A. *A. brassicicola*; B. *B. cinerea*; C. *P. syringae*.

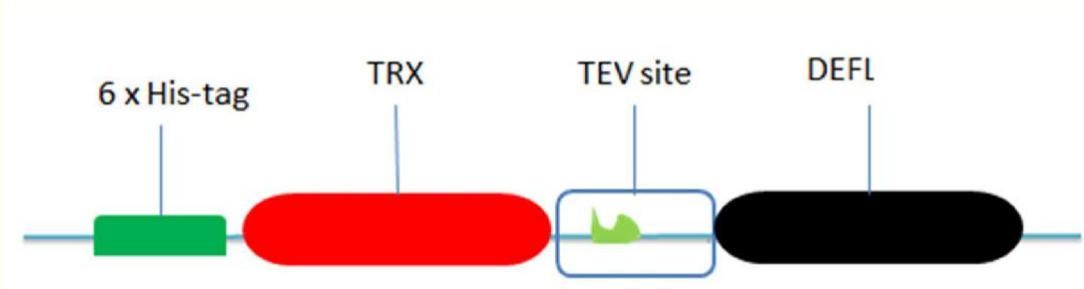

Figure S2. Diagram of the vector for expression of At3g59930 in E. coli. It is based on pETtrx_1a and contains the hexa-histidine tag (green) at the N-terminus for affinity purification.The TRX (red) fusion can be cleaved off from At3g59930 by TEV (tobacco etch virus) protease. TRX= Thioredoxin. DEFL = At3g59930 peptide (black).

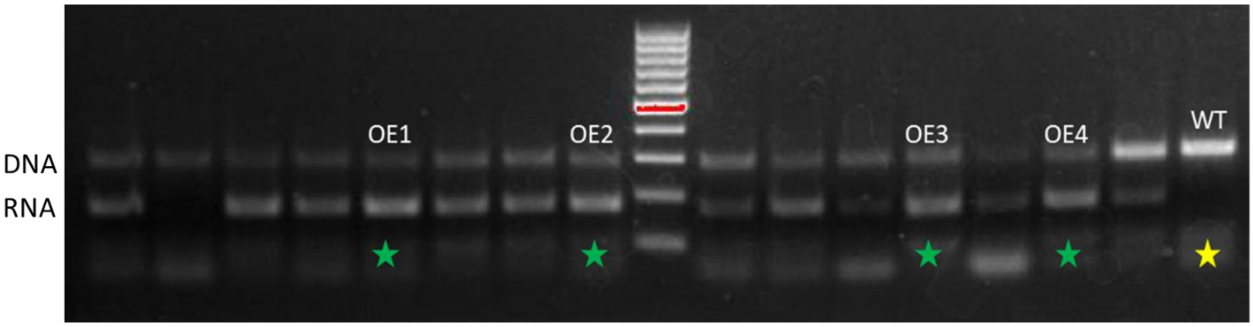

Figure S3. RT-PCR of overexpression lines of *At3g59930*. RT-PCR using RNA isolated from 14 days old seedlings grown on MS medium using gene-specific primers (Table S5). Upper bands indicate DNA while lower bands indicate RNA. Bands with expected size were obtained. Green stars indicate selected overexpression (OE1,2,3 and 4) lines for the production of homozygous lines that are used in downstream experiments. Yellow star indicates wild-type (WT). The ladder used was a 100 bp ladder**.**
